# Community-Based BMI Screening for Overweight and Obesity in Adults Aged 35 Years and Older in Malaysia: Regression Discontinuity Analysis

**DOI:** 10.2196/80381

**Published:** 2025-12-17

**Authors:** Wenjin Chen, Zhoutao Zheng, Shiyu Zhang, Mohd Roshidi Ismail, Pascal Geldsetzer, Till Bärnighausen, Chen Wang, Simiao Chen, Tin Tin Su

**Affiliations:** 1 Chinese Academy of Medical Sciences & Peking Union Medical College Beijing China; 2 Heidelberg University Heidelberg, Baden-Württemberg Germany; 3 South East Asia Community Observatory (SEACO), Global Population Health Jeffrey Cheah School of Medicine and Health Sciences Monash University Subang Jaya, Selangor Malaysia; 4 Stanford University California, CA United States; 5 Harvard T.H. Chan School of Public Health Boston, MA United States; 6 Africa Health Research Institute KwaZulu-Natal South Africa

**Keywords:** BMI, screening, regression discontinuity design

## Abstract

**Background:**

Overweight and obesity are major risk factors for numerous chronic diseases, requiring effective prevention and intervention strategies. Community-based BMI screening may enhance awareness of weight status, but its effectiveness remains uncertain.

**Objective:**

This study aimed to rigorously evaluate the long-term causal effects of community-based BMI screening with a light-touch intervention in Malaysia using a regression discontinuity design (RDD).

**Methods:**

Using data from 2 waves (2013 and 2018) of a Malaysian population-based cohort study (N=6561), we applied a sharp RDD to estimate the causal effects of community-based BMI screening on health outcomes for individuals near the BMI threshold. Participants were aged 35 years or older and completed both follow-ups. The exposure was BMI screening with a light-touch intervention, including height and weight measurement, feedback on results, and referral card distribution. Main outcomes were BMI, blood pressure, and random blood glucose 5 years post intervention, along with health behaviors, health care use, and mental health status.

**Results:**

BMI screening and intervention showed no significant impact on BMI after 5 years (0.4 kg/m², 95% CI –0.2 to 0.9, *P*=.16). Results remained robust after adjusting for covariates (eg, 0.4 kg/m², 95% CI –0.1 to 0.9 with age and sex; 0.5 kg/m², 95% CI –0.1 to 1.0 with demographic covariates) and modifying functional forms (0.4 kg/m², 95% CI –0.2 to 1.1 with quadratic specification). Robustness was also confirmed across different bandwidths, placebo tests, “donut” RDD, and when treating age as either a continuous or categorical variable. Interaction analysis revealed almost no substantial heterogeneity effects. Mechanism analysis and secondary outcomes indicated no significant effects on health behaviors (including smoking, physical activity, diet, and sedentary behavior), health care use (screening, diagnosis, and medication treatment of hypertension and diabetes), mental health outcomes (anxiety, depression, and stress levels), or cardiovascular risk factors (systolic blood pressure, diastolic blood pressure, random blood glucose; eg, systolic blood pressure showed a nonsignificant change of 0.2, 95% CI –3.5 to 4.0 mm Hg). These findings should be interpreted cautiously, as this study was sufficiently powered to detect larger, clinically meaningful changes but may have lacked power to identify more modest effects.

**Conclusions:**

This study is the first to assess the causal effects of population-based BMI screening on long-term health outcomes in a Southeast Asian population. The findings suggest that merely informing individuals of their overweight or obese status and implementing light-touch interventions are insufficient to significantly reduce BMI or drive sustained behavior change. Nonetheless, the results do not exclude the possibility of short-term effects, and more frequent or sustained light-touch interventions may still be effective. Future studies should design more intensive interventions and include larger sample sizes.

## Introduction

Overweight and obesity are significant public health concerns and major risk factors for a range of chronic conditions such as hypertension, diabetes, dyslipidemia, cardiovascular disease (CVD), and certain types of cancer [1,2]. As of 2020, an estimated 2.2 billion adults worldwide were classified as overweight or obese, a number projected to reach 3.3 billion by 2035, representing more than 54% of the global adult population [3]. In Malaysia, the situation is particularly alarming, with approximately 54.4% of adults being overweight or obese as of 2023, marking a 10% increase since 2011 [4]. This trend imposes substantial health and economic burdens, increasing health care costs and reducing quality of life [5,6]. Given the urgency and enormity of the issue, effective prevention and intervention are needed to mitigate the growing burden of overweight and obesity.

One key barrier to effective weight management is the lack of awareness regarding personal weight status [7]. Many individuals are unaware of their BMI or fail to accurately perceive their weight, particularly those classified as overweight or obese [8]. Accurate weight perception is crucial, as studies suggest that individuals who recognize themselves as overweight are more likely to attempt weight loss [9-14]. However, the evidence linking weight perception to actual health behaviors and outcomes is mixed. While some research indicates that perceived overweight motivates healthier behaviors, such as increased physical activity or healthier eating [15], other studies report no association or even negative effects [16,17]. These inconsistencies highlight a critical need for effective interventions to address weight misperception and support health behavior changes.

Community-based BMI screening programs have been proposed as one such approach, aiming to raise awareness of weight status, the overall effectiveness of such programs in improving BMI outcomes remains uncertain. Some studies report significant reductions in BMI and related health risks following screening interventions [18,19], while others find no measurable impact [20,21]. Screening alone may be insufficient to drive significant weight loss if participants lack the resources, motivation, or sustained support to act on the feedback [22]. Moreover, prior studies lacked robust causal inference methodologies and were constrained by short follow-up periods, thereby restricting their ability to comprehensively evaluate the long-term effectiveness of BMI screening interventions.

This study uses a regression discontinuity design (RDD) to rigorously evaluate the causal effects of a community-based BMI screening program conducted in Segamat, Johor, Malaysia. RDD is a quasi-experimental approach that provides robust causal inference in observational settings by exploiting a standard threshold that determines intervention assignment. This method leverages the fact that individuals just above and just below the threshold are likely to be similar in all respects except for their assignment to the intervention group, effectively mimicking a randomized controlled trial. By comparing outcomes near this threshold, RDD isolates the impact of the intervention from other confounding factors. This study evaluates the impact of BMI screening on BMI for over 5 years, alongside secondary outcomes such as systolic blood pressure, diastolic blood pressure, and random blood glucose, which are other CVD risk factors. Additionally, mechanisms of change were analyzed, including health behaviors, mental well-being, and health care use. Subgroup analyses were also conducted to explore heterogeneity across different population groups.

## Methods

### Study Setting

This study analyzed data from the Southeast Asia Community Observatory (SEACO) Health and Demographic Surveillance System [23]. Located in Segamat, Johor, Malaysia, SEACO monitors 5 subdistricts, Bekok, Chaah, Gemereh, Jabi, and Sungai Segamat, of the district’s 11 subdistricts. The surveillance system spans an area of approximately 1250 square kilometers, comprising nearly 13,000 households and a population of about 40,000. A baseline census was conducted from 2012 to 2017, followed by 2 large-scale health surveys (HR) in 2013 and 2018. These surveys included residents aged 5 years and older identified during the baseline census. Participants from the 2012 baseline census were invited to participate in the 2013 health survey (HR 2013), while those from the 2017 census were invited to participate in the 2018 health survey (HR 2018). Exclusion criteria included individuals not enumerated in the baseline census, as well as those who were bedridden, mentally incapacitated, or unable to provide informed consent.

The analysis included participants from HR 2013 and HR 2018. This study’s population was restricted to individuals aged 35 years and older in 2013, as this age group was eligible for biometric assessments and is considered at high risk for chronic conditions. Although height and weight were collected from all individuals aged 5 years and above, the analytic dataset only included participants aged 35 years and older because the planned analysis initially focused on blood pressure, which was measured only in this age group. Among the 13,769 eligible participants from HR 2013, a total of 7024 were successfully followed up on in HR 2018.

### Recruitment Procedures

Field data collection teams consisted of 15-20 members, including a project coordinator, field supervisors, and trained data collectors. Household visits involved explaining this study’s objectives, addressing participant inquiries, and obtaining written informed consent. Data collection comprised structured questionnaires, screening tests, and physical measurements. During the data collection period, households that did not respond were revisited up to 3 times. If no contact was established after 3 attempts, the household was classified as “not at home (nonresponse).” Reasons for nonparticipation were systematically documented only for individuals who explicitly declined participation. A unique SEACO ID was assigned to each participant at their initial contact, facilitating the integration of demographic and health data across multiple surveys and supporting robust longitudinal monitoring.

### Ethical Considerations

This study was approved by the Monash University Human Research Ethics Committee (2018-13142-45226). All participants were provided with information about this study’s purpose and procedures and gave written informed consent before enrollment. Participant privacy and confidentiality were strictly protected: all data were collected and stored in deidentified form on secure servers, and the research team only accessed anonymized datasets during the analysis phase. Participants did not receive any financial or other forms of compensation for their participation. No personally identifiable information or images are included in this manuscript or the supplementary material.

### BMI Measurement and Intervention

Trained field workers measured weight and height using a portable electronic scale with an integrated height sensor (Patient Focus, model GBS-721). Participants were barefoot and wore light clothing during the measurement. BMI was calculated as weight (kg) divided by the square of height (m).

This study used the following approach: all participants were notified of their BMI results by field workers after screening. Individuals with a BMI of ≥25, indicating overweight or obesity, were referred to clinics due to the association between overweight and obesity and an increased risk of CVDs [2]. These individuals are also more likely to have comorbid conditions such as hypertension or hyperglycemia, which warrant further medical evaluation. No additional interventions, follow-ups, or reminders were provided beyond the referral card. With out-of-pocket expenses at local government clinics being minimal (at RM 1 per visit, a currency exchange rate of RM1=US $0.11 was applicable), patients could access full medical consultations, including all necessary examinations and basic laboratory tests [24]. Referral card holders visiting primary care facilities were evaluated and referred by physicians as needed. The “light-touch” intervention involved BMI screening, result communication, and referral card provision, without incorporating incentives, health education, or prescribed time allocations for any activities.

### Study Design

#### About RDD

The RDD offers a robust framework for estimating causal effects by leveraging thresholds within continuous assignment variables to determine intervention assignment. Interventions may be administered deterministically (where individuals on 1 side of the threshold uniformly receive the intervention, termed a “sharp RDD”) or probabilistically (where intervention likelihood differs across the threshold, known as a “fuzzy RDD”) [25-27]. This study used a sharp RDD, as referral cards were distributed based on predefined thresholds, to assess the causal effects of community-based BMI screening. Baseline BMI recorded in 2013 served as the continuous assignment variable. Overweight and obesity were defined using a BMI cutoff of ≥25 kg/m² [28]. Referral card issuance based on these thresholds indicated the need for follow-up health interventions. The findings highlight the intention-to-treat effects of BMI screening, reflected in changes in BMI observed in 2018.

The robustness of RDDs depends on the assumption that potential outcomes are continuous around the threshold. This requires comparability between individuals immediately above and below the threshold, except for their probability of receiving the intervention [29]. A key issue is whether the assignment variable was manipulated. For instance, field workers might record inflated BMI values for participants close to the threshold to increase the number of referral cards distributed. To investigate this, we analyzed the smoothness of the assignment variable at the threshold using a histogram analysis and a formal statistical test developed by Cattaneo et al [30]. This test applies a local polynomial density estimator without binning to detect potential manipulation [30]. Another issue is confounding factors that may be associated with both the threshold and the outcomes. For age to be a true confounder in this RDD, it would need to change abruptly at the threshold, which is unlikely given the smooth distribution of age around BMI thresholds. Even though younger individuals might be more likely to fall below the threshold and older individuals above it, age is likely to vary continuously near the threshold, thereby satisfying the continuity assumption of the RDD. To ensure this assumption holds, we evaluated covariate continuity near the threshold by comparing demographic, socioeconomic, and health behavior variables on either side. Significant differences were identified using chi-square or Fisher exact tests. Where differences existed, we applied the method by Imbens and Lemieux [29], using placebo tests to check for discontinuities in covariates, treating them as outcome variables.

#### Covariates

While the inclusion of covariates is not mandatory for unbiased causal inference in RDD, they play a crucial role in correcting imbalances in characteristics near the threshold in small samples and improving the accuracy of causal effect estimates [31]. For our analysis, adjustments were made for demographic characteristics (age, gender, marital status, and race), socioeconomic factors (education, income, and occupation), and lifestyle behaviors (smoking, alcohol consumption, and physical activity). The covariates were incorporated using an additive-separable and linear-in-parameters specification, maintaining a shared intercept across treated and control groups [31]. To promote transparency and robustness, we initially presented the unadjusted regression discontinuity (RD) estimates, followed by those adjusted with various combinations of covariates. Physical activity was assessed using the Global Physical Activity Questionnaire, with 600 metabolic equivalent of task–minutes or more classified as meeting the World Health Organization’s recommended activity level [32]. Smoking was defined as having ever smoked at least 1 entire cigarette. Alcohol consumption was defined as having consumed alcohol within the past 30 days.

### Statistical Methods

We evaluated the effect of the 2013 overweight and obesity screening on 2018 outcomes using local linear regression, following the methodology of Imbens and Lemieux [29]. Linear models were selected instead of higher-order polynomials to mitigate overfitting and reduce bias in effect estimation [33]. The inclusion of quadratic terms was not statistically significant at the 5% level, suggesting no improvement in model accuracy with higher-order polynomials. Nonetheless, visual inspection highlighted a potential nonlinear association between the assignment variable and the outcomes. To address this, quadratic terms were incorporated as part of a robustness check, and findings were presented for both local linear and quadratic RDDs. A triangular kernel function was used in all models to give greater importance to data points closer to the threshold.

A regression model was fitted for BMI during the main analysis:

*Y_i_*=*α*_0_+*α*_1_*Above_i_*+*α*_2_(*BMI_i_*–25)+*α*_3_*Above_i_*(*BMI_i_*–25)+*X_i_κ*+*v_i_*

*Y_i_* represents the BMI of an individual *i* in 2018. The variable *Above_i_* is set to 1 for individuals whose BMI was at least 25 kg/m² in 2013. *BMI_i_* denotes the BMI of an individual *i* in 2013, while *X_i_* represents the covariate of the individual *i* in 2013. The parameter *α*_1_ captures the average causal effects of community-based screening on BMI in 2018. *α*_2_ indicates the marginal change in BMI in 2018 for individuals whose BMI was below 25 kg/m² in 2013. For individuals with a BMI at or above 25 kg/m² in 2013, the combined effect is given by *α*_2+_*α*_3_. For the evaluation of secondary outcomes, including systolic blood pressure, diastolic blood pressure, and random blood glucose in 2018, similar methodologies were applied as those used for the primary outcome analysis.

In RDDs, selecting the appropriate bandwidths—defining the range around the threshold for fitting local polynomials—is a critical methodological decision. Bandwidths influence the analytical framework, as well as the accuracy and reliability of estimated effects. Narrow bandwidths often provide better model fit by restricting the analysis to observations close to the threshold, where relationships tend to approximate linearity. However, this advantage comes at the expense of increased variance due to the smaller sample size, a challenge described as the “bias-variance tradeoff.” To mitigate this issue, we used the optimal bandwidth selection method introduced by Calonico, Cattaneo, and Titiunik [34] to determine suitable bandwidths for our analysis. We conducted sensitivity analyses to evaluate the robustness of our conclusions by recalculating the primary estimates using different bandwidth settings. In the main analysis, the optimal bandwidth for BMI was 2.9 kg/m². Additionally, we examined the heterogeneity of causal effects across demographic and socioeconomic categories, such as age, sex, marital status, race, education, occupation, and perceived economic status.

Building on the primary impact evaluation, we carried out additional RD analyses to assess the broader effects of the intervention on health behaviors, mental well-being, and health care use. Specifically, we investigated changes in physical activity levels, sedentary behavior, smoking, and nutrition patterns. Psychological outcomes, such as anxiety, depression, and stress, were evaluated using the validated Malay version of the Depression, Anxiety, and Stress Scale-21 [35,36]. Health care use was defined as self-reported information on past screening, diagnosis, and medication treatment for hypertension and diabetes. To analyze binary outcome variables, we applied a modified Poisson regression model within the RD framework to estimate risk ratios, which provide an intuitive measure for interpreting binary outcomes [37]. Specifically, the RD framework used BMI in 2013 as the running variable to determine treatment assignment. Observations within a predefined bandwidth around the cutoff of 25 kg/m² were included in the analysis, with triangular kernel weights applied. The regression models accounted for the treatment effect, the linear trend of the running variable, and their interaction, as described below:

*log*(*Y_i_*)=*α*_0_+*α*_1_*Above_i_*+*α*_2_(*BMI_i_*–25)+*α*_3_*Above_i_*(*BMI_i_*–25)+*ε_i_*

The outcome variable *Y_i_* represents a binary indicator derived from health-related outcomes in 2018, modeled using a log-link function. The variable *Above_i_* is defined as 1 for individuals whose BMI in 2013 was at least 25 kg/m², and 0 otherwise. The parameters α₁, α₂, and α₃ capture the treatment effect, the marginal trend below the cutoff, and the interaction between treatment and BMI deviations from the cutoff, respectively. By integrating the RD framework with modified Poisson regression, this approach enabled the estimation of local treatment effects and risk ratios, which are directly interpretable for binary outcomes based on BMI [38-41]. All analyses and graphical representations were carried out using R software (version 4.2.2; R Foundation).

## Results

### Sample Characteristics

**Figure 1:**
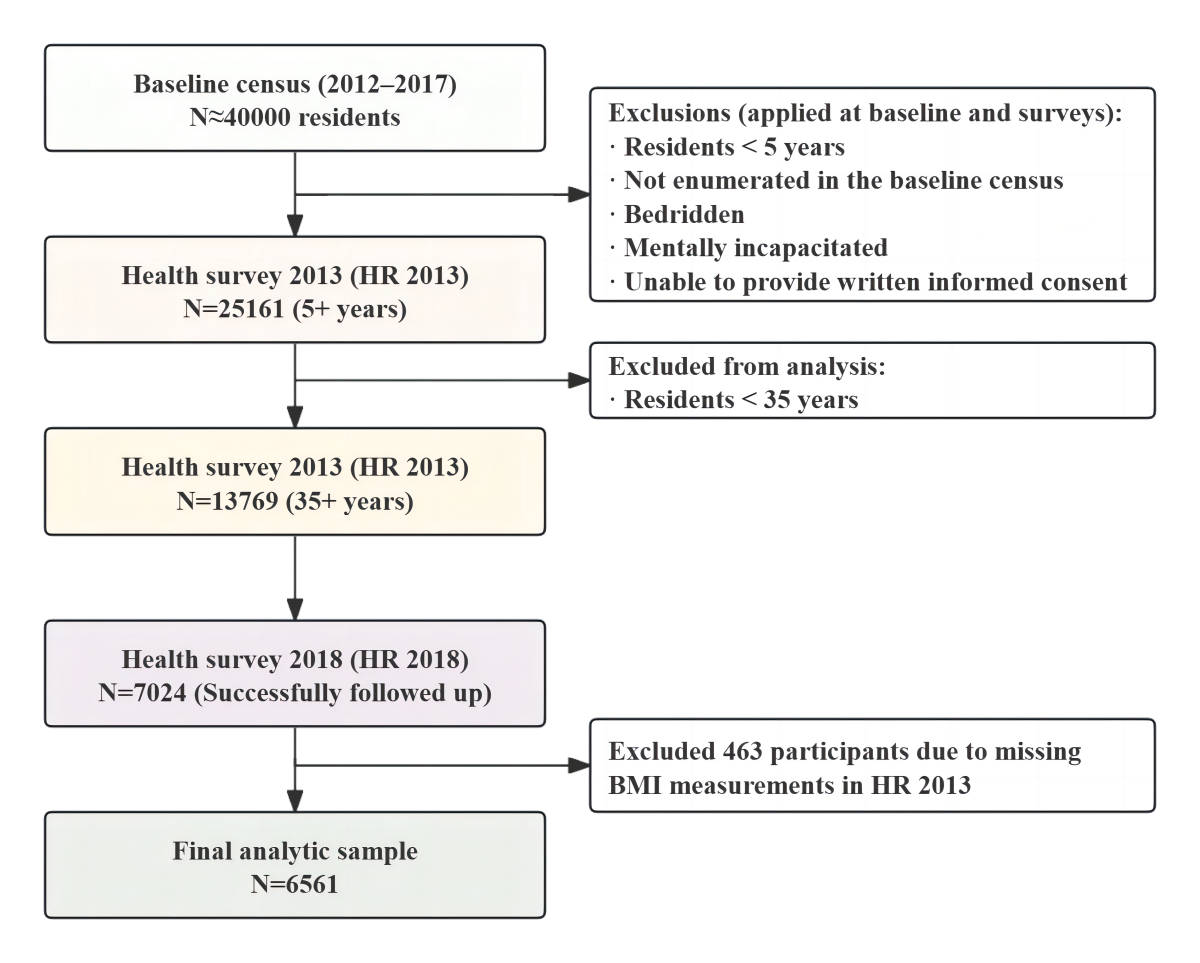
A flow diagram for participant selection. HR: health survey.

A total of 7024 individuals were initially included in the sample. We excluded 463 (6.6%) participants due to missing BMI measurements in 2013, leaving 6561 participants (Figure 1). Given the high attrition between 2013 and 2018, we formally tested whether follow-up rates were discontinuous around the BMI cutoff. As shown in Section S1 in Multimedia Appendix 1, no statistically significant discontinuity was detected, and the results were robust to alternative bandwidth choices (50% and 150% of the optimal bandwidth). Table 1 provides an overview of the full sample and the groups within the optimal BMI bandwidths. The demographic, socioeconomic, and behavioral health traits are comparable across both groups. Specifically, 34% (n=2239) of participants are aged 50-59 years, 40% (n=2641) are male, 85% (n=5529) are married, 47% (n=2947) possess a primary education level, and 14% (n=891) are smokers.

**Table 1:** Demographic, socioeconomic, and health behavior characteristics of adults ≥35 years, Segamat, Malaysia, overall sample and within bandwidth samples^a^.

Characteristics			Full sample (n=6561), n (%)		Sample within the MSE^b^-optimal bandwidth (n=3024), n (%)
**Demographic**					
	**Age group (years)**				
		35 to 39	527 (8)	193 (6.4)	
		40 to 49	1516 (23)	666 (22)	
		50 to 59	2239 (34)	1012 (33)	
		60 to 69	1636 (25)	799 (26)	
		70 and above	643 (9.8)	354 (12)	
	Male		2641 (40)	1351 (45)	
	Married		5529 (85)	2556 (85)	
	**Race**				
		Malay	4198 (64)	1854 (61)	
		Chinese	1497 (23)	801 (26)	
		Indian	760 (12)	329 (11)	
		Orang Asli	75 (1.1)	24 (0.8)	
		Other	31 (0.5)	16 (0.5)	
**Socioeconomic**					
	**Education level**				
		No formal education	193 (3.1)	98 (3.4)	
		Primary	2947 (47)	1368 (48)	
		Secondary	2892 (46)	1310 (45)	
		Tertiary	235 (3.7)	104 (3.6)	
	**Personal gross monthly income (RM)^c^**				
		<1000	2370 (45)	1073 (43)	
		1000-1999	1764 (33)	858 (34)	
		2000-2999	667 (13)	327 (13)	
		3000 and above	521 (9.8)	241 (9.6)	
	**Occupation**				
		Paid-employee	1575 (24)	747 (25)	
		Self-employed	1230 (19)	623 (21)	
		Homemaker	2576 (39)	1065 (35)	
		Not working	717 (11)	347 (11)	
		Pensioners and other	450 (6.9)	236 (7.8)	
**Health behavior**					
	Ever smoked		891 (14)	432 (14)	
	Drink alcohol		168 (2.6)	87 (2.9)	
	Exercise		916 (14)	420 (14)	

^a^Exercise represents the proportion of participants meeting the World Health Organization’s recommendation for physical activity (≥600 metabolic equivalent of task–minutes per week), assessed using the Global Physical Activity Questionnaire.

^b^MSE: mean squared error.

^c^A currency exchange rate of RM1=US $0.11 was applicable.

### Manipulation of Assignment Variable

**Figure 2:**
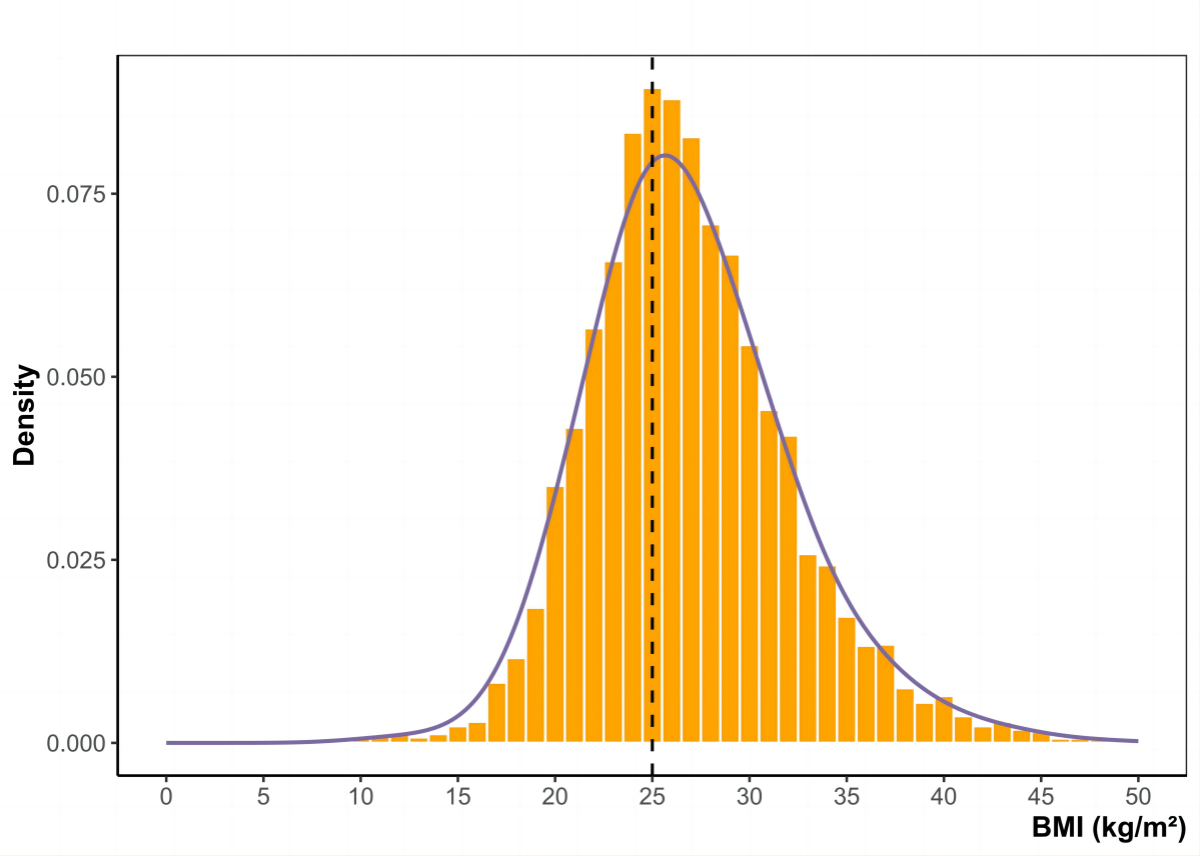
Density of baseline BMI distribution among adults ≥35 years, Segamat, Malaysia, 2013. The dashed line represents the threshold of 25 kg/m². Participants with a BMI exceeding this limit were given a referral card by field workers, indicating that they had reached a critical level of being overweight or obese. No additional interventions were provided. The sample size is 6561.

The RDD necessitates that the assignment variable (BMI in 2013) remains free from manipulation near the threshold. Figure 2 presents a histogram of the assignment variable to assess any indications of manipulation. No visual evidence of bunching was detected near the threshold, providing evidence against potential interference in screening measurements by field workers. Additionally, the formal test introduced by Cattaneo et al [30] confirmed the null hypothesis of smooth density around the threshold, further supporting the absence of manipulation in the assignment variable (Section S2 in Multimedia Appendix 1).

### Continuity of Participants’ Observed Characteristics Around Threshold

The RDD assumes no other interventions impact outcomes near the BMI thresholds. Table 2 presents the demographic, socioeconomic, and health behavior characteristics of individuals just above and below the threshold within the optimal bandwidth. Most characteristics are balanced; however, slight imbalances were detected for sex and age. These discrepancies were identified through preliminary chi-square and Fisher exact tests, which were used as screening tools to detect potential imbalances near the threshold. For covariates showing significant differences in these preliminary tests (eg, sex and age), we conducted placebo tests using the framework by Imbens and Lemieux [29]. These placebo tests treated the covariates as outcomes to directly assess potential discontinuities at the threshold. The results confirmed no significant discontinuities, thereby supporting the continuity assumption and the validity of the RDD (Section S3 in Multimedia Appendix 1).

**Table 2:** Proportions of covariates above and below BMI discontinuity thresholds within optimal bandwidths, adults ≥35 years, Segamat, Malaysia, 2013^a^.

Characteristics				BMI					Difference in proportions (n=3024)
				≥25 kg/m² (n=1568, 95% CI)		<25 kg/m² (n=1456, 95% CI)			
**Demographic**									
	**Age group (years)**								
		35 to 39	5.9 (4.8-7.2)		6.9 (5.7-8.4)		–1.1		
		40 to 49	22.3 (20.3-24.5)		21.7 (19.7-23.9)		0.6		
		50 to 59	34.5 (32.2-36.9)		32.3 (30.0-34.8)		2.2		
		60 to 69	27.0 (24.8-29.2)		25.8 (23.6-28.1)		1.2		
		70 and above	10.3 (8.9-11.9)		13.2 (11.5-15.0)		–2.9*		
	Male		42.8 (40.4-45.3)		46.7 (44.2-49.3)		–3.9*		
	Married		84.6 (82.7-86.3)		85.3 (83.4-87.0)		–0.7		
	**Race**								
		Malay	61.5 (59.1-63.9)		61.1 (58.5-63.5)		0.5		
		Chinese	25.4 (23.3-27.6)		27.7 (25.4-30.0)		–2.3		
		Indian	11.5 (10.0-13.2)		10.2 (8.8-11.9)		1.2		
		Orang Asli	0.9 (0.5-1.5)		0.7 (0.4-1.3)		0.2		
		Other	0.7 (0.4-1.3)		0.3 (0.1-0.8)		0.4		
**Socioeconomic**									
	**Education level**								
		No formal education	3.4 (2.6-4.5)		3.4 (2.6-4.5)		0.0		
		Primary	48.7 (46.1-51.2)		46.2 (43.6-48.9)		2.4		
		Secondary	44.1 (41.6-46.6)		47.0 (44.4-49.7)		–3.0		
		Tertiary	3.9 (3.0-5.0)		3.3 (2.5-4.4)		0.6		
	**Personal gross monthly income (RM)^b^**								
		<1000	43.0 (40.3-45.7)		42.9 (40.1-45.7)		0.1		
		1000-1999	33.0 (30.5-35.6)		35.7 (33.1-38.5)		–2.7		
		2000-2999	13.8 (12.0-15.8)		12.3 (10.6-14.3)		1.5		
		3000 and above	10.2 (8.7-12.0)		9.0 (7.6-10.8)		1.2		
	**Occupation**								
		Paid-employee	23.6 (21.5-25.7)		26.0 (23.8-28.3)		–2.4		
		Self-employed	21.3 (19.4-23.4)		19.9 (17.9-22)		1.5		
		Homemaker	35.3 (32.9-37.7)		35.3 (32.9-37.8)		0.0		
		Not working	11.9 (10.4-13.7)		11.0 (9.5-12.7)		0.9		
		Pensioners and other	7.9 (6.6-9.3)		7.8 (6.5-9.3)		0.1		
	**Health behavior**								
		Smoke	14.2 (12.5-16.0)		14.6 (12.8-16.5)		–0.4		
		Drink alcohol	3.1 (2.3-4.1)		2.7 (2.0-3.7)		0.4		
		Exercise	13.9 (12.3-15.7)		13.9 (12.2-15.8)		0.0		

^a^*P<.05. The bandwidth for BMI is 22.1 to 27.9 kg/m².

^b^A currency exchange rate of RM1=US $0.11 was applicable.

### Impact Estimates

**Figure 3:**
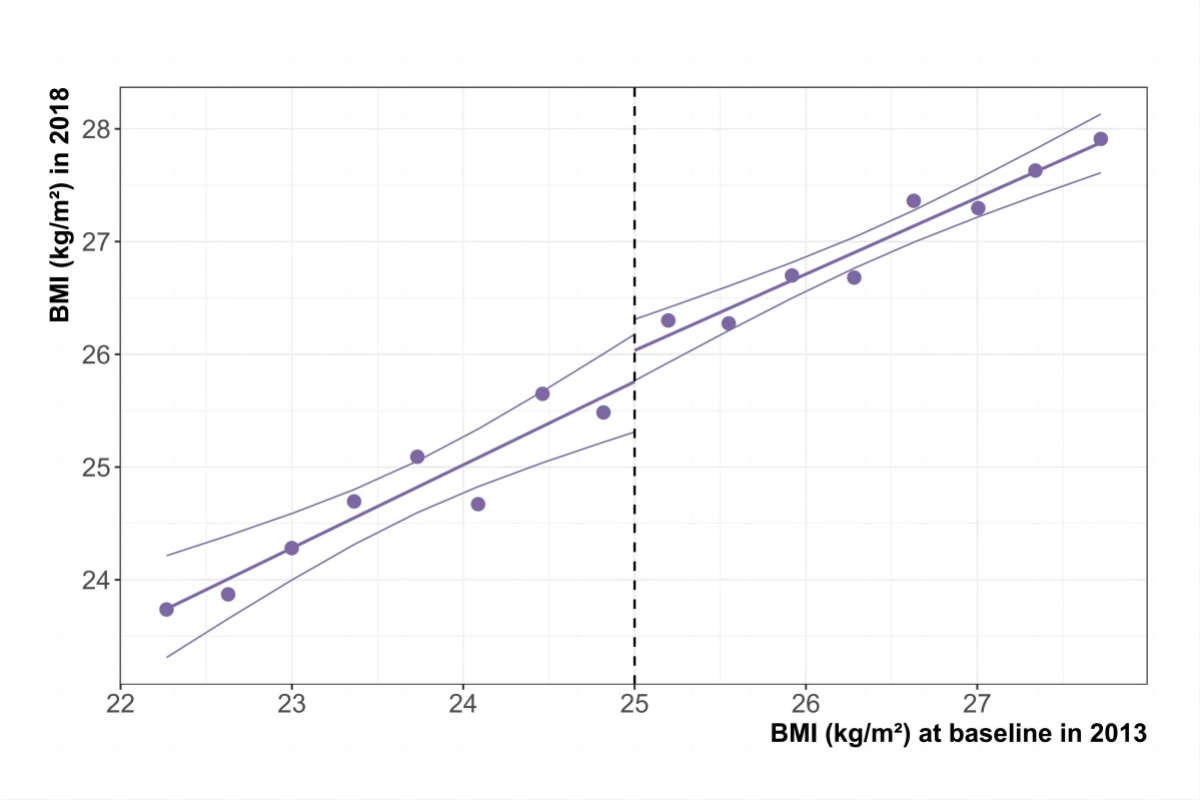
Five-year change in BMI (2013-2018) within optimal bandwidths, adults ≥35 years, Segamat, Malaysia. The dashed line marks the threshold at which field workers provided a referral card, without implementing further interventions. Thick lines depict the linear fit, while thin lines illustrate the 95% CIs. The total sample consists of 6561 individuals, including 3024 within the optimal bandwidth for BMI.

Figure 3 depicts the effect of the assignment variable (2013 BMI) on the outcome variables (2018 BMI) within the optimal bandwidth, with the threshold represented by a dashed line. No notable shifts in BMI were identified across the discontinuity, implying that the community’s overweight or obesity screening conducted in 2013 may not have significantly influenced BMI in 2018.

Table 3 outlines the regression estimates of the local causal effect of community screening. The analysis showed that community screening did not significantly reduce BMI (0.4 kg/m², 95% CI –0.2 to 0.9, *P*=.16) by 2018. After controlling for demographic, socioeconomic, and health behavior variables, the impact of screening remained insignificant. Consistent results were obtained using local quadratic regression approaches.

**Table 3:** Regression discontinuity estimates with 95% CIs for the effect of 2013 community-based BMI screening on 2018 BMI outcomes, adults ≥35 years, Segamat, Malaysia.

Impact of screening on BMI (kg/m²)			Without covariates		With age and sex		With demographic covariates		With demographic and social covariates		With demographic, social, and behavioral covariates
**Local linear**											
	BMI	0.4 (–0.2 to 0.9)		0.4 (–0.1 to 0.9)		0.5 (–0.1 to 1.0)		0.3 (–0.3 to 1.0)		0.3 (–0.3 to 1.0)	
	*P* value	.16		.15		.08		.31		.30	
**Local quadratic**											
	BMI	0.4 (–0.2 to 1.1)		0.4 (–0.2 to 1.1)		0.6 (–0.1 to 1.2)		0.4 (–0.4 to 1.1)		0.4 (–0.3 to 1.1)	
	*P* value	.19		.17		.09		.31		.30	

To assess heterogeneity in the effects of community screening, we conducted interaction regression analyses. Although the overall interaction by age group was not statistically significant, subgroup analyses suggested an effect among individuals aged 50-59 years (–1.3, 95% CI –2.2 to –0.4 kg/m²; P for heterogeneity=0.01). In contrast, no substantial heterogeneity and interaction were detected based on sex, race, marital status, education level, income, or occupation (Section S4 in Multimedia Appendix 1).

The sample consists of individuals falling within the optimal bandwidth around the 25 kg/m² threshold, using baseline BMI as the assignment variable. Each cell reports the coefficient from a separate regression, using a triangular kernel function that assigns greater weight to observations nearer to the threshold. The models in the second column do not include covariates; the third column controls only for age and sex, the main covariates with baseline imbalances, to help isolate their influence and enhance the transparency of robustness checks; the fourth column controls for demographic covariates (age, sex, marital status, and race); the fifth column incorporates social covariates (education, occupation, and self-reported monthly income); and the sixth column includes behavioral covariates (smoking, drinking, and physical activity).

### Secondary Outcomes and Mechanism Exploration

Additional RD analyses indicate that the intervention had no significant impact on health behaviors, such as smoking habits, physical activity, sedentary behavior, and daily vegetable and fruit intake. Mental health outcomes, including anxiety, depression, and stress levels, were also unaffected. Similarly, no significant changes were observed in cardiovascular risk factors, such as systolic blood pressure, diastolic blood pressure, and random blood glucose, as well as the use of health services for hypertension and diabetes screening, diagnosis, and medication treatment (Section S5 in Multimedia Appendix 1).

### Robustness Check

**Figure 4:**
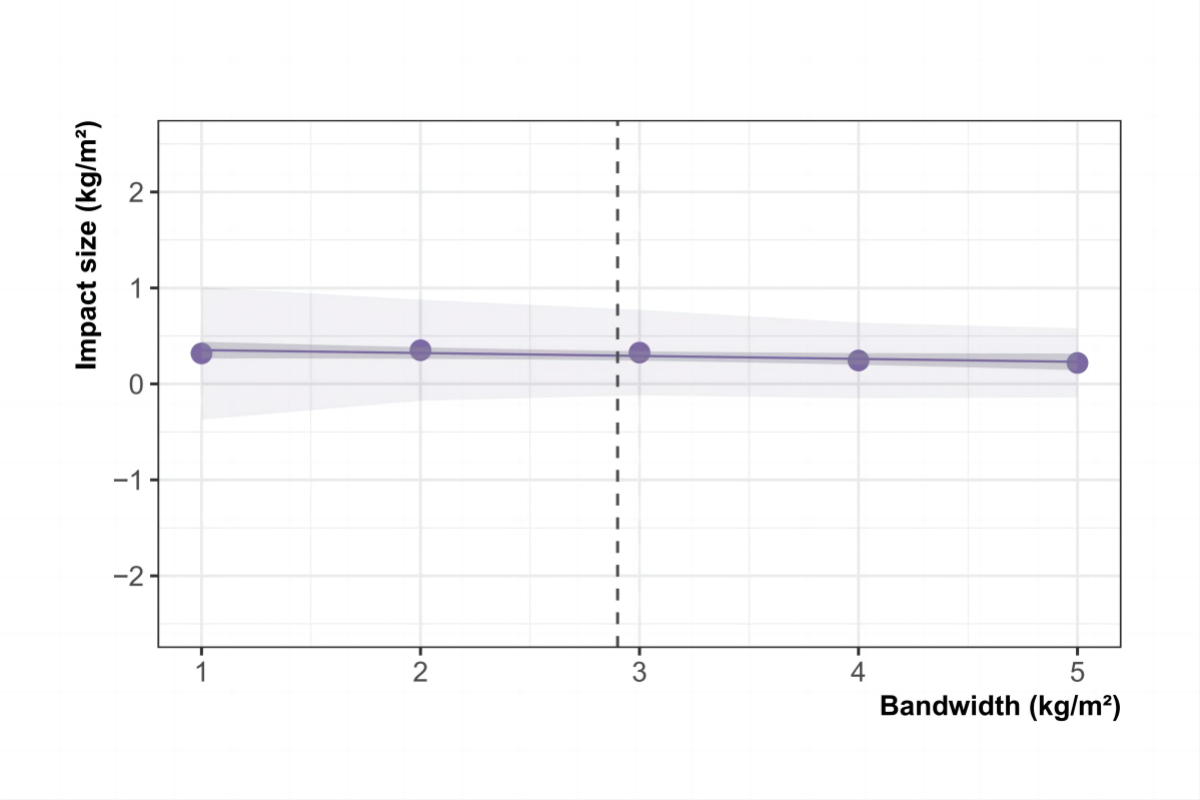
Impact estimates for BMI across different bandwidths in adults ≥35 years, Segamat, Malaysia, 2013-2018.

We examined the robustness of our findings by evaluating the impact estimates for BMI across different bandwidths, placebo tests, and “donut” RDD. First, alongside the optimal bandwidth, we included bandwidths of 50%, 80%, 120%, and 150% to track potential changes in the estimates (Figure 4). The analysis indicated that as the bandwidth changed, the impact estimates for BMI consistently approached 0. Second, Section S6 in Multimedia Appendix 1 presents RDD estimates based on placebo tests using alternative BMI cutoff values. Specifically, when applying alternative cutoffs at 24, 24.5, 25.5, and 26, the estimates were all statistically insignificant (eg, −0.18, 95% CI −0.77 to 0.41 at BMI 24). At the true cutoff of 25, the estimate was also statistically insignificant, consistent with the overall null findings of the main analysis. Third, we further conducted a “donut” RDD by systematically excluding observations within 0.5, 1.0, and 1.5 BMI units around the cutoff. This strategy addresses potential concerns that observations located very close to the threshold may be subject to nonrandom sorting or measurement error. The results, presented in Section S7 in Multimedia Appendix 1, are qualitatively similar to the main estimates, thereby ruling out local manipulation at the threshold as a source of bias. Additionally, we tested the robustness of our results by treating age as a continuous variable rather than categorizing it into groups. This alternative specification captures finer variations in age and avoids potential biases introduced by arbitrary categorization. The results remained consistent with those obtained using the categorical approach, further supporting the robustness of our conclusions (Section S8 in Multimedia Appendix 1). These further corroborate our conclusion that community screening did not have a significant effect on BMI after 5 years.

## Discussion

### Principal Findings

This study examined the long-term impact of a population-based BMI screening intervention on health outcomes among adults aged 35 years and older in Malaysia. Using an RDD, the analysis provided causal evidence that the intervention did not result in a significant reduction in BMI over 5 years. This finding remained consistent across various model specifications, including adjustments for covariates, alternative functional forms, bandwidth modifications, placebo tests, and “donut” RDD. While the screening informed participants of their overweight and obesity status, the absence of measurable BMI changes underscores the limitations of screening as a standalone strategy for promoting sustained weight management. At the same time, this is the first study in Southeast Asia to rigorously evaluate the causal effects of such a simple, low-cost, and low-intensity program, with the Malaysian setting, which is marked by high primary care coverage and low costs despite being a middle-income country, and a predominantly rural population with relatively low education and income levels, providing important contextual relevance and international comparability.

### Insignificant Reduction in BMI

There are several plausible explanations for the insignificant reduction in BMI observed 5 years after the intervention. First, the intervention was low intensity, consisting solely of BMI measurement and the distribution of referral cards, without structured follow-up, behavioral counseling, or actionable guidance, which likely limited its effectiveness. This “light-touch” approach does not adequately address the multifaceted drivers of obesity, such as dietary habits, physical activity, socioeconomic constraints, and psychosocial influences. Prior studies have demonstrated that low-intensity interventions are often insufficient to sustain long-term behavior change, particularly when participants lack consistent engagement or access to actionable resources for weight management [38,42]. Second, the 5-year follow-up period may have diminished any short-term effects of the intervention [43]. While initial awareness of weight status may have prompted temporary behavioral changes, the absence of reinforcement likely led participants to revert to pre-existing habits [44]. Moreover, over such an extended period, external factors such as aging, life transitions, or independent health decisions could have influenced participants’ behaviors and health outcomes, further complicating the attribution of any observed effects directly to the intervention. Therefore, we cannot rule out the possibility that short-term effects occurred initially but faded over time, which may not have been captured in the 5-year follow-up. This possibility also suggests that more frequent or sustained light-touch interventions might still be effective in maintaining behavior change. Third, the universal coverage and low out-of-pocket costs of Malaysia’s public health care system likely moderated the intervention’s impact. With the government subsidizing approximately 98% of treatment costs at public health facilities [24,45], individuals identified as overweight or obese could independently access routine care without relying on the program’s referrals. In high-resource health care settings, the referral cards may have limited additional value compared to settings where health care access is restricted. Fourth, participant characteristics may have further constrained the intervention’s effectiveness. This study’s population, drawn predominantly from rural areas and characterized by relatively low educational attainment, may have faced barriers such as limited health literacy and reduced understanding of the long-term risks associated with elevated BMI [46]. Additionally, the risk of adverse metabolic outcomes increases from approximately 35 years of age, and the estimated treatment effect may have been confounded by cumulative age-related changes over the 5-year follow-up. Prior evidence indicates that longer cumulative exposure to obesity elevates the risk of type 2 diabetes and other cardiometabolic conditions [47]. Finally, behavioral responses among participants with a healthy BMI (<25 kg/m²) may also have contributed. Being informed of a normal weight could have been interpreted as reassurance, leading some individuals to relax weight control or adopt riskier behaviors. Such compensatory responses, if present, may have biased the estimated impact downward. Although our data do not allow us to directly test this mechanism, acknowledging this possibility is important for interpreting the null findings.

### Heterogeneity Analysis and Mechanism Exploration

The heterogeneity analysis revealed no significant differences in BMI outcomes across demographic and socioeconomic subgroups, including age, gender, and educational attainment. Similarly, no significant differences were observed when comparing individuals just above and below the BMI threshold, suggesting that the intervention’s effect was uniformly limited across subpopulations. Further exploration into the mechanisms underlying this limited impact revealed no significant changes in associated behaviors, such as fruit and vegetable consumption, physical activity, or smoking. These findings underscore the intervention’s inability to address the behavioral determinants of weight management. Cardiovascular risk factors, including waist circumference, blood pressure, and blood glucose levels, also showed no significant improvement, consistent with findings from other studies [19]. Notably, receiving a referral card did not negatively affect the mental health of individuals with overweight or obesity, an important consideration for the intervention’s safety profile. While some studies have reported that weight notification may increase the risk of mental health conditions [48,49], this study found no significant effects on anxiety, depression, or stress. Despite the lack of significant changes, health care use increased modestly among individuals near the BMI threshold, particularly in screening and diagnosis rates for conditions such as hypertension and diabetes. As shown in Section S4 in Multimedia Appendix 1, participants around the BMI threshold exhibited similar patterns of health behaviors but notable increases in screening, diagnosis, and, in some cases, treatment rates for hypertension and diabetes between 2013 and 2018. These findings suggest that individuals near the threshold became more attentive to their health status, which, combined with Malaysia’s universal coverage and low out-of-pocket costs, facilitated greater health care use. However, these increases did not translate into measurable changes in BMI outcomes, as the comprehensive health system likely moderated the incremental impact of this light-touch intervention. A critical factor to consider is the specificity and utility of BMI screening as a public health tool. Compared to more targeted screenings, such as blood pressure or glucose testing, BMI measurement is simpler but less directly linked to actionable health risks. While BMI screening effectively categorizes individuals based on weight status, it does not provide immediate clinical priorities or tailored recommendations, potentially diminishing its perceived urgency among participants.

### Limitations

This study has several limitations. Although data were generated from the whole community in 5 subdistricts of Segamat, it does not represent a nationwide representative sample [23]. Consequently, the findings may not be generalizable to more urbanized populations or to individuals with higher socioeconomic status, higher income, better education, and greater health literacy, who may respond differently to such light-touch interventions. The 2-wave design, lacking short-term postintervention data, restricts the ability to observe dynamic changes in health behaviors or outcomes. Furthermore, the RD approach estimates causal effects only for individuals near the BMI threshold, reducing applicability to broader population groups, which may respond differently to the screening intervention. Additionally, we cannot exclude modest effect sizes due to the width of our CIs, suggesting relatively low statistical power. For BMI screening, our power calculations indicate that this study was sufficiently powered (85%) to detect larger effect sizes (eg, τ=0.8 kg/m²) within the optimal bandwidth but underpowered to detect clinically meaningful smaller changes. Clinical guidelines typically define a ≥5% reduction in body weight as clinically meaningful because of consistent improvements in glycemia, blood pressure, and lipid profiles [50]. As BMI is proportional to weight for fixed height, this corresponds to an approximate BMI reduction of 0.05*baseline BMI. Within our analytic bandwidth (22.1-27.9 kg/m^2^), a 5% weight loss translates into a BMI decrease of about 1.1-1.4 kg/m^2^. Thus, while our study was likely sufficiently powered to detect such clinically meaningful individual-level effects, it was underpowered to detect smaller but potentially important shifts in population health, as seen in prior policy evaluations [51,52]. Lastly, reliance on self-reported data for secondary outcomes may introduce recall and social desirability biases, and the overall follow-up rate of about 51% raises concerns about attrition bias. Attrition differed by baseline BMI category, with lower follow-up among participants with BMI <25 compared to those with BMI ≥25 (46.1% vs 54.6%; difference=–8.5%, 95% CI –10.2% to –6.8%, *P*<.001), suggesting potential bias. However, the RDD focuses on local comparisons around the cutoff rather than overall representativeness, reducing dependence on complete follow-up. Moreover, continuity tests of follow-up rates at the BMI cutoff (using optimal, 50%, and 150% bandwidths) showed no discontinuities. Additional robustness checks, including bandwidth modifications, “donut” RDD and placebo tests, yielded consistent null results, indicating that selective attrition near the threshold is unlikely to bias our estimates. Thus, while attrition bias cannot be completely ruled out, current evidence suggests it is unlikely to drive the main findings. Despite these limitations, this study provides critical evidence on the challenges and potential of BMI screening, underscoring the need for more intensive, multifaceted strategies to enhance its impact on population health.

### Health Policy Implications

The findings of this study underscore the need for integrating BMI screening into comprehensive public health strategies to effectively address overweight and obesity in middle-income settings such as Malaysia. While BMI screening is a low-cost and scalable tool, its impact as a standalone intervention is limited. This highlights the necessity of combining it with complementary measures to achieve meaningful and sustained health outcomes. To enhance the effectiveness of BMI screening, policy makers should incorporate tailored behavioral interventions such as personalized counseling, health education, and goal-setting. These components can equip individuals with actionable strategies and sustained motivation to adopt healthier lifestyles. Additionally, integrating follow-up mechanisms, such as regular check-ins or digital health tools, can reinforce behavioral changes and mitigate regression over time.

Structural barriers to weight management must also be addressed. Improving access to affordable healthy foods, creating safe spaces for physical activity, and promoting health literacy are critical policy measures to create supportive environments for weight management. Leveraging Malaysia’s extensive public health care infrastructure can ensure that affordable, accessible interventions reach individuals identified as at-risk through BMI screening.

### Conclusions

Merely informing individuals with overweight or obesity about their weight status is insufficient to achieve significant reductions in BMI or foster sustained behavior change. More intensive interventions are required to support individuals in adopting and maintaining healthy behaviors. They should also address structural barriers to effective obesity prevention and treatment following community-based BMI screening. Nonetheless, our findings do not exclude the possibility of short-term effects. This suggests that more frequent or sustained light-touch interventions might still be effective in promoting lasting benefits. Future studies should incorporate larger sample sizes to enhance the ability to detect modest but clinically meaningful effect sizes.

## Appendix

Multimedia Appendix 1 Extended regression discontinuity design analyses: manipulation and continuity tests, heterogeneity, secondary outcomes and mechanisms, and robustness checks.

## Abbreviations

CVD: cardiovascular disease
HR: health survey
RD: regression discontinuity
RDD: regression discontinuity design
SEACO: Southeast Asia Community Observatory

## Data Availability

The individual-level datasets generated and analyzed during this study are not publicly available to avoid the risk of participant reidentification but are available from the corresponding author on reasonable request.
