# Supplementary material for: Community-Based BMI Screening for Overweight and Obesity in Adults Aged 35 Years and Older in Malaysia: Regression Discontinuity Analysis
Source: JMIR Public Health Surveill. 2025 Dec 17;11:e80381. doi: 10.2196/80381 (PMC12756664; doi:10.2196/80381)
Supplement: Multimedia Appendix 1 [file publichealth_v11i1e80381_app1.docx]

**Supplementary Material**

[A: Continuity of participants’ follow-up rate around the threshold in the 2013 baseline data 1](#_Toc30587)

[B: Data manipulation test 2](#_Toc20429)

[C: Continuity of participants’ observed characteristics around the threshold 2](#_Toc7189)

[D: Heterogeneity among groups 3](#_Toc9463)

[E: Second outcomes and mechanism analysis 5](#_Toc14546)

[F: Placebo tests 8](#_Toc19118)

[G: “Donut” RDD 8](#_Toc25094)

[H: Robustness test 9](#_Toc21925)

This document serves as a supplement to the article " **Community-Based BMI Screening for Overweight and Obesity in Adults Aged 35 Years and Older in Malaysia: Regression Discontinuity Analysis**" offering an expanded analysis and additional context to support the research conclusions.

## A: Continuity of participants’ follow-up rate around the threshold in the 2013 baseline data

**Table S1** reports results from continuity tests of participants’ follow-up rates at the 2013 baseline, using BMI=25 as the cutoff. These tests assess whether attrition between 2013 and 2018 introduces discontinuities around the threshold. Robustness was further checked using bandwidths equal to 50% and 150% of the optimal bandwidth.

**Table S1. RDD estimates testing the continuity of participants’ follow-up rate around the BMI cutoff in the 2013 baseline data**

| **Bandwidth multiplier** | **BMI as running variable** | | |
| --- | --- | --- | --- |
|  | **Coefficient (95% CI)** | **P value** | **Optimal bandwidth** |
| 0.5 | -0.05 (-0.15 to 0.04) | 0.263 | 1.97 |
| 1 | -0.06 (-0.13 to 0.00) | 0.065 | 3.94 |
| 1.5 | -0.05 (-0.10 to 0.01) | 0.089 | 5.91 |

Note: Estimates are obtained from local linear RDD regressions using the rdrobust package. A triangular kernel was applied with bandwidths equal to the optimal value, as well as 50% and 150% of the optimal bandwidth. Robust standard errors were clustered on the running variable. The results show no evidence of discontinuities in follow-up rates around the BMI cutoff.

## B: Data manipulation test

Beyond visually examining the continuity of BMI distribution at the threshold, we employed the local polynomial density estimator developed by Cattaneo et al.^1^ This approach avoids reliance on pre-binning and was implemented with the optimal bandwidth to identify any signs of data manipulation near the threshold. The resulting hypothesis test yielded a p-value of 0.97, which did not reject the null hypothesis of smooth density at the threshold. This result further supports the assumption that the field staff adhered to rigorous data collection standards without manipulation.

## C: Continuity of participants’ observed characteristics around the threshold

To investigate the continuity of participants' observed characteristics near the threshold, we first conducted chi-square and Fisher’s exact tests to screen for potential imbalances in covariates. These tests identified slight imbalances in sex and age near the threshold. Chi-square and Fisher’s exact tests were used solely as preliminary screening tools to detect potential differences in covariates and inform subsequent analyses. For the covariates identified as imbalanced (e.g., sex and age), we applied the Imbens and Lemieux framework to conduct regression discontinuity-based placebo tests.^2^ These tests treated the imbalanced covariates as outcomes, using BMI as the running variable, to directly assess whether significant discontinuities occurred at the threshold. The results, as shown in **Table S2**, confirmed no significant discontinuities for these covariates at the threshold. This finding supports the continuity assumption of the regression discontinuity design, confirming the validity of the model.

**Table S2. Estimating the continuity of observed characteristics around the threshold for BMI using control variables as outcomes**

| **Characteristics** | **BMI as running variable** | |
| --- | --- | --- |
|  | **Coefficient (95% CI)** | **P value** |
| Age group: 70 and above | 0.02 (-0.08 to 0.13) | 0.644 |
| Sex: Male | -0.09 (-0.27 to 0.09) | 0.327 |

## D: Heterogeneity among groups

To investigate potential heterogeneity in the influence of 2013 BMI screening on 2018 BMI outcomes across various groups, we introduced a group indicator variable along with an interaction term.

The subsequent regression model was employed within the optimal bandwidth for local linear regression:

$$\begin{matrix} Y_{i} & =\alpha_{0}+\alpha_{1}Above_{i}+\alpha_{2}({BMI}_{i}-25)+\alpha_{3}Above_{i}({BMI}_{i}-25)+\alpha_{4}G_{i}+\alpha_{5}G_{i}Above_{i}+v_{i} \end{matrix}$$

Here:

- $Y_{i}$ represents individual $i$'s BMI in 2018.
- $Above_{i}$ is an indicator equal to 1 if individual $i$ had a BMI of at least 25 kg/m² in 2013.
- ${BMI}_{i}$ denote individual $i$'s BMI measurements in 2013.
- $G_{i}$ captures the group effect, while the coefficient $\alpha_{5}$ indicates potential group-based heterogeneity.

This structure allows us to examine whether the effects of screening vary across groups.

**Table S3. Regression discontinuity estimates of the impact of BMI screening in 2013 on BMI outcome in 2018**

| **Interaction terms** | **Impact of screening on BMI with baseline BMI as the running variable (kg/m²) (95% CI)** | **P for heterogeneity** | **P for interaction** |
| --- | --- | --- | --- |
| **Age group** |  |  |  |
| 40 to 49 | -0.84 (-1.81 to 0.13) | 0.091 | 0.077 |
| 50 to 59 | -1.28 (-2.22 to -0.35) | 0.007* |  |
| 60 to 69 | -0.8 (-1.76 to 0.15) | 0.098 |  |
| 70 and above | -0.97 (-2.04 to 0.1) | 0.075 |  |
| **Male** | -0.1 (-0.54 to 0.34) | 0.650 | 0.650 |
| **Race** |  |  |  |
| Chinese | 0.5 (-0.01 to 1) | 0.056 | 0.194 |
| Indian | 0.28 (-0.45 to 1) | 0.455 |  |
| Orang Asli | 1 (-1.5 to 3.51) | 0.433 |  |
| Other | 2.42 (-0.83 to 5.68) | 0.144 |  |
| **Married** | -0.24 (-0.85 to 0.38) | 0.453 | 0.453 |
| **Education** |  |  |  |
| Primary | -0.03 (-1.28 to 1.22) | 0.964 | 0.651 |
| Secondary | -0.24 (-1.49 to 1.02) | 0.711 |  |
| Tertiary | 0.43 (-1.26 to 2.12) | 0.62 |  |
| **Income** |  |  |  |
| RM1000-RM1999 | 0.5 (-0.03 to 1.04) | 0.065 | 0.097 |
| RM2000-RM2999 | -0.27 (-1.01 to 0.47) | 0.479 |  |
| RM3000 and above | -0.26 (-1.1 to 0.57) | 0.536 |  |
| **Occupation** |  |  |  |
| Self-employed | 0.55 (-0.1 to 1.2) | 0.099 | 0.525 |
| Homemaker | 0.4 (-0.17 to 0.97) | 0.165 |  |
| Not working | 0.41 (-0.36 to 1.19) | 0.297 |  |
| Pensioners and other | 0.35 (-0.55 to 1.24) | 0.445 |  |

**Notes:** The sample includes individuals with BMI within the optimal bandwidth around the 25 kg/m² threshold. Regressions use a triangular kernel function to assign greater weight to observations closer to the threshold. Each cell in the table presents the coefficient from a separate regression. *P<0.05. The column “P for heterogeneity” shows the p-value for the subgroup-specific interaction coefficient (testing whether the effect for that level differs from the reference), while the column “P for interaction” gives the p-value from a Wald test of the joint significance of all interaction terms for that subgroup variable (testing overall effect modification).

## E: Second outcomes and mechanism analysis

In addition to the primary outcomes, we examined secondary outcomes to explore potential mechanisms underlying the effects of BMI screening. As shown in **Tables S4–S7**, no significant differences were observed in health behaviors or mental health outcomes around the BMI threshold. However, healthcare utilization showed modest increases between 2013 and 2018 among individuals near the threshold, particularly in screening and diagnosis rates for hypertension and diabetes, and in some cases treatment rates. These patterns suggest that while BMI screening did not directly alter lifestyle or psychological factors, it may have contributed to a greater likelihood of engaging with healthcare services over time.

**Table S4. Regression discontinuity estimates of the impact of BMI screening in 2013 on health behaviors, healthcare utilization and mental health in 2018**

|  | **Coefficient (95% CI)** |
| --- | --- |
| **Health behaviors** |  |
| Smoking | 1.04 (0.74, 1.45) |
| Exercise | 1.05 (0.87, 1.27) |
| Meals | 0.19 (-0.35, 0.72) |
| Fruit | 0.04 (-0.05, 0.12) |
| Vegetable | 0.04 (-0.05, 0.12) |
| Sedentary | -0.23 (-0.59, 0.14) |
| **Healthcare utilization** |  |
| Screened in hypertension | 1.09 (0.82, 1.44) |
| Diagnosed in hypertension | 1.15 (0.76, 1.72) |
| Treated in hypertension | 1.06 (0.88, 1.27) |
| Screened in diabetes | 1.41 (0.95, 2.1) |
| Diagnosed in diabetes | 1.45 (0.66, 3.25) |
| Treated in diabetes | 1.09 (0.82, 1.44) |
| **Mental health** |  |
| Depression | 1.76 (0.57, 5.76) |
| Anxiety | 1.36 (0.62, 3) |
| Stress | 1.18 (0.15, 8.93) |

**Notes:** Estimates for Meals, Fruit, Vegetable and Sedentary are regression coefficients (β) because they are continuous variables. Estimates for all other variables, including Smoking, Exercise, Healthcare Utilization, and Mental Health (Depression, Anxiety, Stress), are relative risks (RR) as they are binary outcomes.

**Table S5. Regression discontinuity estimates of the impact of BMI screening in 2013 on CVD risk factors in 2018**

|  | **RBG** | | **BMI** | | **SBP** | | **DBP** | |
| --- | --- | --- | --- | --- | --- | --- | --- | --- |
|  | β | P | β | P | β | P | β | P |
| **Model 1** | 0.3 (-0.4 to 1.0) | 0.42 | 0.4 (-0.2 to 0.9) | 0.16 | 0.2 (-3.5 to 4.0) | 0.90 | 0.3 (-1.8 to 2.3) | 0.79 |
| **Model 2** | 0.3 (-0.5 to 1.2) | 0.45 | 0.4 (-0.2 to 1.1) | 0.19 | 0.8 (-3.3 to 5.0) | 0.70 | 0.6 (-1.6 to 2.8) | 0.62 |
| **Model 3** | 0.3 (-0.5 to 1.0) | 0.48 | 0.5 (-0.1 to 1.0) | 0.08 | 0.2 (-3.4 to 3.8) | 0.93 | 0.5 (-1.6 to 2.6) | 0.65 |
| **Model 4** | 0.3 (-0.6 to 1.2) | 0.52 | 0.6 (-0.1 to 1.2) | 0.09 | 0 (-3.8 to 3.8) | 1.00 | 0.8 (-1.5 to 3.0) | 0.50 |
| **Model 5** | 0.4 (-0.4 to 1.3) | 0.30 | 0.3 (-0.3 to 1.0) | 0.31 | 0.5 (-3.5 to 4.5) | 0.80 | 0.5 (-1.9 to 2.8) | 0.68 |
| **Model 6** | 0.4 (-0.5 to 1.4) | 0.37 | 0.4 (-0.4 to 1.1) | 0.31 | 1.1 (-3.5 to 5.6) | 0.64 | 0.7 (-1.9 to 3.3) | 0.59 |
| **Model 7** | 0.4 (-0.4 to 1.2) | 0.35 | 0.3 (-0.3 to 1.0) | 0.30 | 0.3 (-3.6 to 4.3) | 0.87 | 0.5 (-1.9 to 2.8) | 0.69 |
| **Model 8** | 0.4 (-0.6 to 1.4) | 0.42 | 0.4 (-0.3 to 1.1) | 0.30 | 1.0 (-3.6 to 5.6) | 0.68 | 0.7 (-1.9 to 3.2) | 0.61 |

**Notes**: RBG, random blood glucose; BMI, body mass index; SBP, systolic blood pressure; DBP, diastolic blood pressure. Models 1, 3, 5, and 7 use local linear regression, while Models 2, 4, 6, and 8 use local quadratic regression. Covariates include none (Models 1-2), demographic (Models 3-4), demographic and social (Models 5-6), and demographic, social, and behavioral (Models 7-8).

**Table S6: Hypertension healthcare utilization in 2013 and 2018**

| **Year** | **Screened** | | | **Diagnosed** | | | **Treated** | | |
| --- | --- | --- | --- | --- | --- | --- | --- | --- | --- |
|  | BMI≥25 | BMI<25 | Mean Difference | BMI≥25 | BMI<25 | Mean Difference | BMI≥25 | BMI<25 | Mean Difference |
| 2013 | 66.9 (64.5-69.2) | 59.8 (57.3-62.3) | 7.1 | 36.8 (33.7-40) | 22.4 (19.7-25.4) | 14.4 | 27.8 (25-30.8) | 16.5 (14.1-19.2) | 11.3 |
| 2018 | 93.9 (92.6-95) | 92.2 (90.7-93.4) | 1.7 | 51.5 (47.8-55.1) | 48.4 (44.3-52.4) | 3.1 | 18.6 (15.9-21.7) | 20.8 (17.7-24.3) | -2.2 |

**Table S7: Diabetes healthcare utilization in 2013 and 2018**

| **Year** | **Screened** | | | **Diagnosed** | | | **Treated** | | |
| --- | --- | --- | --- | --- | --- | --- | --- | --- | --- |
|  | BMI≥25 | BMI<25 | Mean Difference | BMI≥25 | BMI<25 | Mean Difference | BMI≥25 | BMI<25 | Mean Difference |
| 2013 | 58.6 (56.1-61) | 51.9 (49.3-54.4) | 6.7 | 21.8 (19.2-24.6) | 13 .0  (10.9-15.5) | 8.8 | 6.9 (5.4-8.8) | 2.9  (2.0-4.3) | 4.0 |
| 2018 | 88.9 (87.2-90.4) | 87.8 (86.1-89.4) | 1.1 | 40 .0  (35.9-44.2) | 32.7 (28.3-37.5) | 7.2 | 10.3 (8-13.3) | 9.8 (7.3-13.2) | 0.5 |

## F: Placebo tests

**Table S8** reports placebo tests based on alternative BMI cut-off values to assess the robustness of the RDD estimates. These tests are intended to detect potential spurious discontinuities at neighboring thresholds.

**Table S8. RDD estimates of the impact of 2013 community-based BMI screening on 2018 BMI outcomes at alternative cut-offs (placebo tests)**

| **Cutoff** | **BMI as running variable** | | |
| --- | --- | --- | --- |
|  | **Coefficient (95% CI)** | **P value** | **Optimal bandwidth** |
| 24.0 | -0.18 (-0.77 to 0.41) | 0.547 | 2.49 |
| 24.5 | 0.12 (-0.49 to 0.74) | 0.698 | 2.76 |
| 25.0 | 0.38 (-0.15 to 0.9) | 0.164 | 2.91 |
| 25.5 | -0.10 (-0.62 to 0.43) | 0.712 | 3.16 |
| 26.0 | -0.15 (-0.64 to 0.35) | 0.566 | 3.53 |

**Notes:** The table reports RDD estimates of BMI outcomes at different cut-off values. The alternative cut-offs include 24.0, 24.5, 25.5, and 26.0, in addition to the pre-specified cutoff of 25.0. Each cell reports the coefficient from a separate local linear regression, employing a triangular kernel function that assigns greater weight to observations closer to the threshold. Robust standard errors are clustered on the running variable. No additional covariates were included in these models.

## G: “Donut” RDD

**Table S9** reports results from “donut” RDD specifications, where observations within varying ranges around the cutoff were systematically excluded. This approach mitigates potential concerns about local manipulation or sorting at the threshold.

**Table S9. RDD estimates of the impact of 2013 community-based BMI screening on 2018 BMI outcomes (“Donut” RDD)**

| **Excluding observations near the threshold** | **BMI as running variable** | | |
| --- | --- | --- | --- |
|  | **Coefficient**  **(95% CI)** | **P value** | **Optimal bandwidth** |
| 0 | 0.38 (-0.15 to 0.90) | 0.164 | 2.91 |
| 0.5 | 0.37 (-0.79 to 1.53) | 0.530 | 2.42 |
| 1.0 | 0.97 (-0.86 to 2.81) | 0.297 | 2.34 |
| 1.5 | 1.36 (-1.94 to 4.66) | 0.420 | 2.59 |

**Notes:** Each cell reports the coefficient from a separate local linear regression discontinuity design, estimated using the rdrobust package. A triangular kernel function was applied, assigning greater weight to observations closer to the threshold. Optimal bandwidths were selected using the mserd criterion. Robust standard errors were clustered on the running variable. The “donut” specification excludes observations within 0.5, 1.0, and 1.5 BMI units of the cutoff.

## **H: Robustness test**

**Table S10** shows that our main results remain consistent when age is modeled as a continuous variable rather than using categorical groupings (e.g., 35-44, 45-54, 55-64, 65-74, and 75+ years). This alternative specification leverages the full variability in the data, providing a more precise representation of age effects while confirming the robustness of our findings.

**Table S10. Regression discontinuity estimates with 95% confidence intervals for the effect of 2013 community-based BMI screening on BMI outcomes in 2018.**

| **Impact of screening on**  **BMI (kg/m²)** | **Without covariates** | **With demographic**  **covariates** | **With demographic and**  **social covariates** | **With demographic, social, and**  **behavioural covariates** |
| --- | --- | --- | --- | --- |
| **BMI**  **(local linear)** | 0.4  (-0.2 to 0.9) | 0.5  (0.0 to 1.0) | 0.3  (-0.3 to 1.0) | 0.3  (-0.3 to 1.0) |
| P value | 0.16 | 0.07 | 0.30 | 0.28 |
| **BMI**  **(local quadratic)** | 0.4  (-0.2 to 1.1) | 0.6  (-0.1 to 1.2) | 0.4  (-0.3 to 1.1) | 0.4  (-0.3 to 1.2) |
| P value | 0.19 | 0.08 | 0.30 | 0.28 |

**Notes:** The sample consists of individuals falling within the optimal bandwidth around the 25 kg/m² threshold, using baseline BMI as the assignment variable. Each cell reports the coefficient from a separate regression, employing a triangular kernel function that assigns greater weight to observations nearer to the threshold. The models in the second column do not include covariates; the third column controls for demographic covariates, where age is modeled as a continuous variable alongside sex, marital status, and race; the fourth column incorporates social covariates (education, occupation, and self-reported monthly income); and the fifth column includes behavioral covariates (smoking, drinking, and physical activity).

**Reference**

1. Cattaneo MD, Idrobo N, Titiunik R. A Practical Introduction to Regression Discontinuity Designs: Foundations. Cambridge: Cambridge University Press; 2020.

2. Imbens GW, Lemieux T. Regression discontinuity designs: A guide to practice. *Journal of Econometrics* 2008; **142**(2): 615-35.
